# Progress and Expansion of the National Wastewater Surveillance System, United States, 2020–2024

**DOI:** 10.3201/eid3213.261118

**Published:** 2026-08

**Authors:** Miguella Mark-Carew, Megan Bias, Stacie Reckling, Kaniz Madhobi, Daniel Cornforth, Igor Volkov, Andrew L. Rainey, Benjamin Lorentz, Kaitlin R. Taibl, Lisa P. Oakley, Rachel West, Rory Welsh, Jeffrey W. Mercante, Niketta Womack, Nicole Fehrenbach, Jonathan Yoder

**Affiliations:** Centers for Disease Control and Prevention, Atlanta, Georgia, USA (M. Mark-Carew, M. Bias, K. Madhobi, D. Cornforth, A.L. Rainey, K.R. Taibl, L.P. Oakley, R. West, R. Welsh, J.W. Mercante, N. Womack, N. Fehrenbach, J. Yoder); Chenega Corporation, Anchorage, Alaska, USA (S. Reckling, I. Volkov); Goldbelt Professional Services LLC, Chesapeake, Virginia, USA (B. Lorentz)

**Keywords:** viruses, COVID-19, SARS-CoV-2, public health surveillance, wastewater, wastewater surveillance, wastewater-based epidemiological monitoring, United States

## Abstract

Wastewater surveillance in the United States has evolved from an emergency response initiative into a valuable component of public health infrastructure. During its first 4 years, the Centers for Disease Control and Prevention’s National Wastewater Surveillance System (NWSS) expanded its national testing coverage, suite of pathogen targets, analytics capabilities, data visualizations, and laboratory methods. Collaborations across public, private, and academic sectors continue to drive progress toward standardization of practices, interpretation of wastewater signals, and translation of wastewater surveillance into public health action. This article describes the evolution of NWSS from late 2020 through 2024, highlighting its integration into public health practice and its demonstrated adaptability and scalability as a surveillance system supporting timely, data-driven decision-making to address community-level public health threats.

Wastewater surveillance has become a valuable component of the United States public health infrastructure since late 2020, when the Centers for Disease Control and Prevention (CDC) launched the National Wastewater Surveillance System (NWSS) to coordinate testing and reporting for SARS-CoV-2 in wastewater (*1*,*2*). NWSS is a partnership between state, tribal, local, and territorial public health departments (hereafter referred to as health departments), commercial entities, and academia to track a range of pathogen targets in sewersheds, or the community area contributing wastewater to a sample site. As a relatively new surveillance system, NWSS has quickly transformed from a rapid-response effort into an adaptive surveillance structure that can support long-term infectious disease monitoring. NWSS complements case-based and syndromic surveillance by providing timely insights into infectious disease signals at the community level (*3*).

Since 2020, NWSS has expanded its wastewater sampling network across the continental United States and its territories, growing from 295 sites representing ≈12% of the US population in 2020 to >1,800 sites representing ≈45% of the US population in 2024 (*4*). During that same period, NWSS broadened its scope from a single-pathogen system tracking only SARS-CoV-2 to one that monitors multiple pathogen targets. In 2022, NWSS added testing for monkeypox virus (MPXV) in response to the 2022 global outbreak and has since expanded to additional targets such as influenza A (and subtypes), influenza B, and respiratory syncytial virus (RSV). That growth has been supported by the One CDC Data Platform (1CDP, formerly DCIPHER), alongside improvements in analytic methods and data visualizations (*3*,*5*). The NWSS program has also fostered collaboration across the public and private sector, including with health departments, commercial laboratories, nonprofit entities, and academic institutions to further WS science. This article describes NWSS from its inception through 2024, updating previously published findings (*4*), with an emphasis on advances in system coverage, pathogen monitoring, analytic capabilities, and public health application during 2023 and 2024.

## Materials and Methods

WS data are submitted to NWSS via 1CDP by health departments, CDC commercial contractors, and an academic partner program (*6*). We limited our analyses to calendar years through 2024, encompassing the first 4 years of NWSS implementation and capturing substantial growth in participating sites and pathogen targets. After downloading concentration data on May 4, 2026, we filtered it to include samples collected from September 1, 2020, through December 31, 2024, and categorized data into 3 time periods: January–December 2023, January–December 2024, and September 2020–December 2024. On May 1, 2026, we downloaded and analyzed sequencing metadata corresponding to paired concentration samples available on June 5, 2025. We included wastewater sites with >1 sample collected during each analytic period. Data for other pathogen targets were collected, but our analysis mainly focused on SARS-CoV-2, MPXV, influenza A virus, influenza A(H5) subtype, and RSV, given broad national testing coverage for those pathogens.

We mapped wastewater sampling sites by using the centroid coordinates of the wastewater treatment plant postal (ZIP) codes, stratified by year and number of targets assayed. We assessed county urbanicity by using the US Department of Agriculture 2023 Rural-Urban Continuum Codes (*7*) and calculated counts of NWSS sites by US Census Region (*8*). Assessing data submission timeliness involved parsing dates and excluding records with invalid dates. We used a Python script using Palantir’s Foundry API (https://www.palantir.com/docs/foundry/api/v2) and the pandas library (https://pandas.pydata.org) to scan the metadata of archived submissions to determine start dates for automated 1CDP submissions. We conducted descriptive analyses by using R software version 4.4.0 (The R Foundation for Statistical computing, https://www.r-project.org) and Python version 3.12.4 (https://www.python.org) pandas library version 2.2.2 and geospatial analyses using ArcGIS Pro 3.1.7 (https://www.esri.com). We pulled SARS-CoV-2 wastewater sequencing data and metadata submitted to the National Center for Biotechnology Information (NCBI) by health department and commercial contract partners into 1CDP to determine the dominant variant at each participating wastewater site on the basis of the highest relative lineage abundance from mixed SARS-CoV-2 samples. We processed all sequencing samples by using the CFSAN Wastewater Analysis Pipeline (C-WAP; https://github.com/CFSAN-Biostatistics/C-WAP). This activity was reviewed by CDC and conducted consistent with applicable federal law and CDC policy (see, e.g., 45 C.F.R. part 46.102(l)(2), 21 C.F.R. part 56; 42 U.S.C. §241(d); 5 U.S.C. §552a; 44 U.S.C. §3501 et seq.).

## NWSS Site Coverage and Characteristics

**Figure 1:**
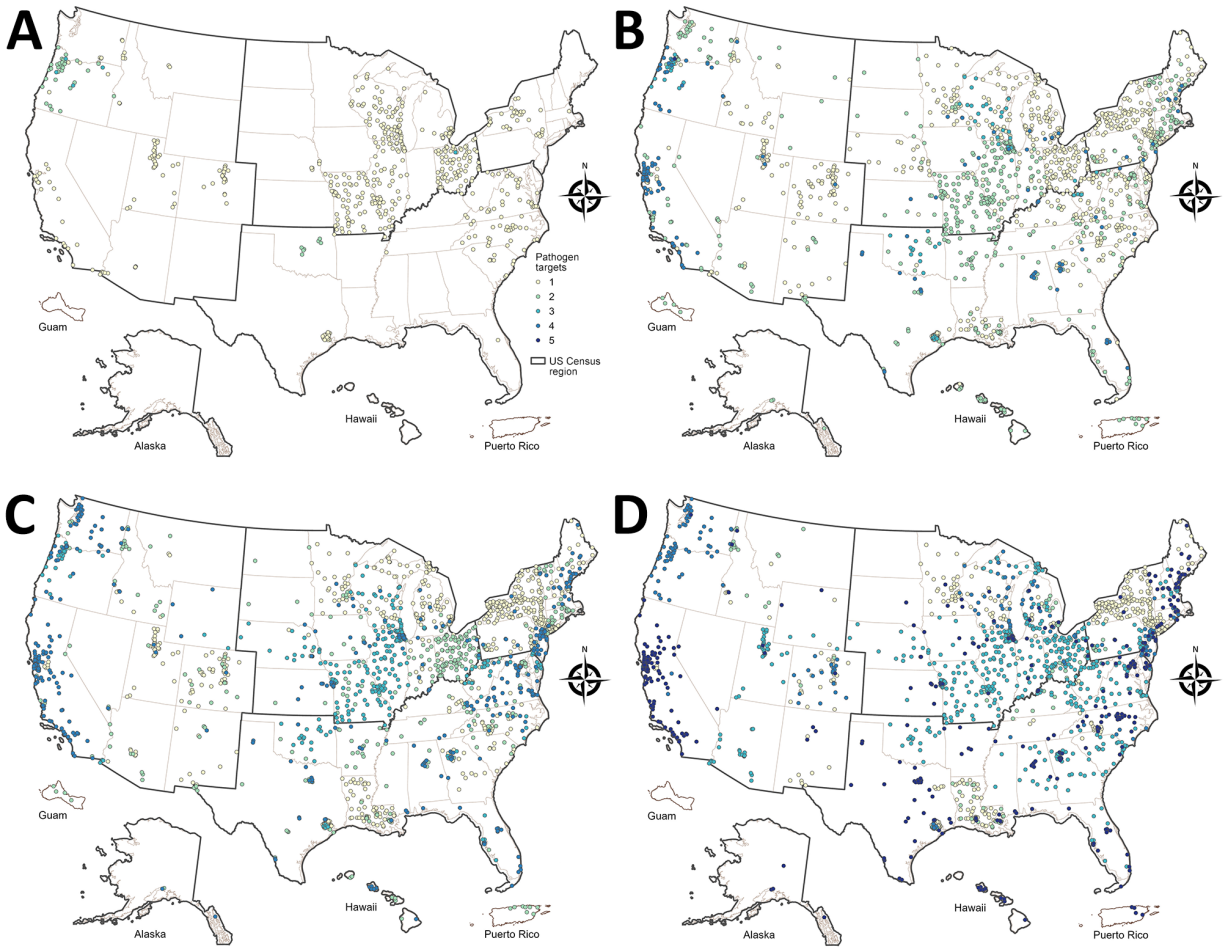
Geographic distribution of National Wastewater Surveillance System testing sites in the United States and territories, September 2020–December 2024. Each site is categorized by the number of viral targets tested during the specified period. Site coverage and viral target testing are shown for September 2020–December 2021 (A), 2022 (B), 2023 (C), and 2024 (D). US Census Regions are available from https://www.census.gov/geographies/mapping-files/time-series/geo/cartographic-boundary.html.

**Figure 2:**
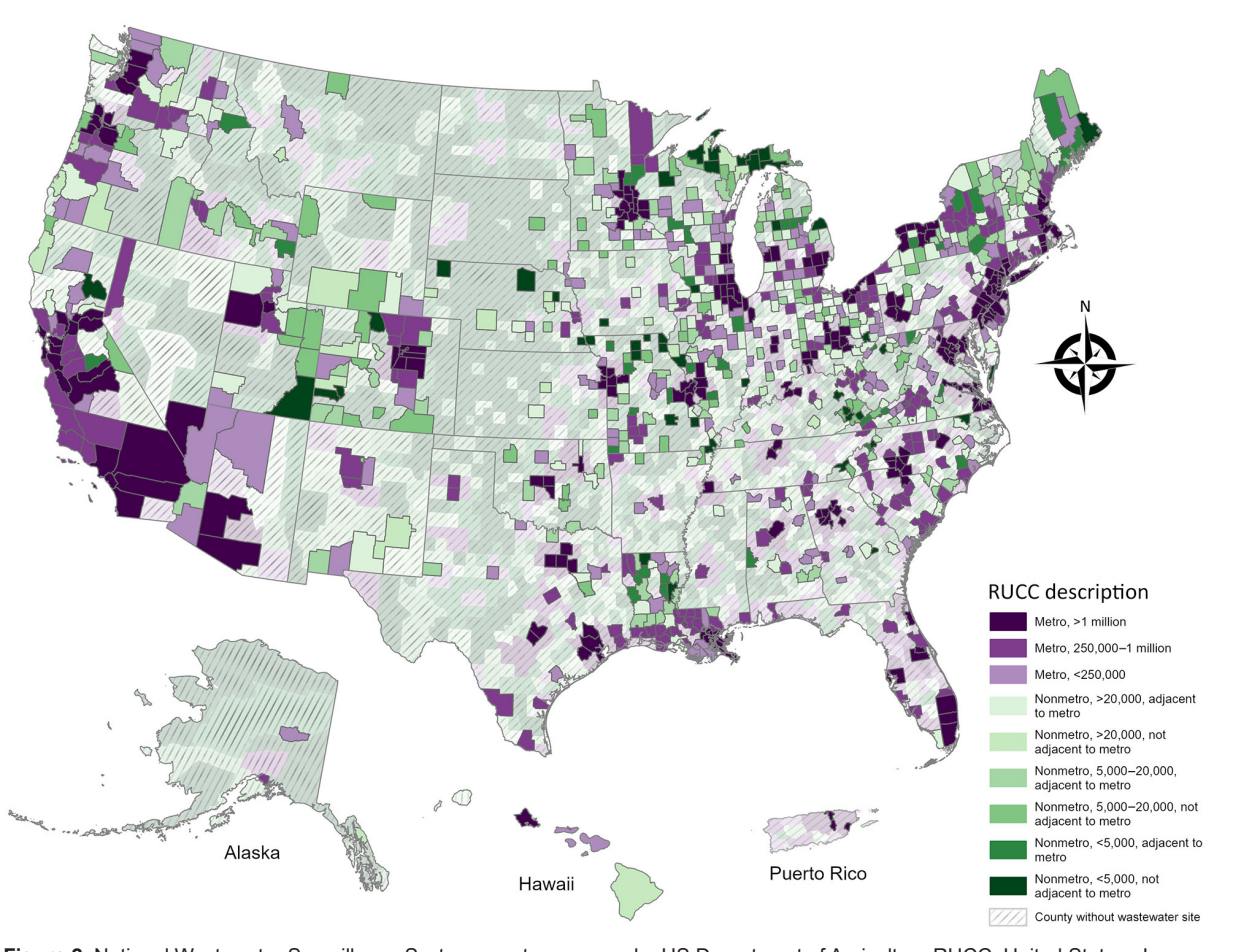
National Wastewater Surveillance System county coverage by US Department of Agriculture RUCC, United States, January 1–December 31, 2024. Counties with >1 wastewater site that reported to NWSS are displayed as bolder colors; muted colors with hatching represent no site coverage. RUCC data were from the 2023 RUCC dataset (https://www.ers.usda.gov/data-products/rural-urban-continuum-codes). Metro, metropolitan; RUCC, Rural–Urban Continuum Code.

NWSS has achieved broad geographic coverage of the United States, representing 178 million persons across 53 states and territories. US population coverage was >45% in both 2023 and 2024 and for the full 2020–2024 period (Table 1). Most wastewater sampling sites were in the South and Midwest US Census Regions across all time periods (Table 1; Figure 1). Wastewater sampling sites were concentrated in highly populated urban counties; in 2023 and 2024, metropolitan counties represented ≈60% of counties served by the NWSS network but 39% of all US counties (Table 1; Figure 2). Approximately 80% of sites were managed by health departments. The number of wastewater sampling sites and counties decreased slightly from 2023 to 2024 (1,967 sites in 2023 to 1,871 sites in 2024 and 1,029 counties in 2023 to 990 counties in 2024).

**Table 1:** Characteristics of sites participating in the National Wastewater Surveillance System, United States, 2023, 2024, and 2020–2024*

Characteristics	2023	2024	2020–2024
States and territories, no.	53	52	53
Counties, no.†	1,029	990	1,180
County RUCC, by population, no. (%)‡			
Metropolitan			
>1 million	274 (27)	245 (25)	280 (24)
250,000–1 million	187 (18)	197 (20)	214 (18)
<250,000	156 (15)	155 (16)	175 (15)
Nonmetropolitan			
Adjacent to metro area >20,000	102 (10)	105 (11)	119 (10)
Not adjacent to metro area >20,000	39 (4)	30 (3)	45 (4)
Adjacent to metro area 5,000–20,000	94 (9)	94 (10)	119 (10)
Not adjacent to metro area 5,000-20,000	78 (8)	75 (8)	96 (8)
Adjacent to metro area <5,000	45 (4)	42 (4)	62 (5)
Not adjacent to metro area <5,000	54 (5)	47 (5)	70 (6)
Sites by US Census region, no.§			
Midwest	705	662	829
Northeast	376	340	404
South	480	509	642
West	393	356	443
Site population			
Persons served by all sites, no. (% of US population), in millions¶	163 (49)	150 (45)	178 (54)
Persons served per site, median (IQR), in thousands	24 (8–77)	24 (8–75)	21 (7–67)
Site characteristics			
Sites, no.	1,967	1,871	2,331
Tribal land sites, no.	7	8	14
Site location			
WWTP	1,672	1,605	1,988
Upstream	295	266	343
Targets tested for at site, no.#			
SARS-CoV-2	1,952	1,847	2,308
Monkeypox virus	650	481	731
Influenza A	870	1,301	1,364
Influenza A(H5)	25	551	551
Respiratory syncytial virus	723	1,215	1,256
Site management, no. (%)**			
Local health department	63 (3)	62 (3)	63 (2)
State health department	1,523 (77)	1,551 (83)	1,853 (79)
Commercial national contractor	397 (20)	188 (10)	493 (21)
Academic partner program	198 (10)	192 (10)	199 (9)
Laboratories testing samples from sites, no.	95	102	139
No. samples collected per site per week, median (IQR)††	2 (1–2)	2 (1–2)	2 (1–2)
Automated concentration data submission, sites (health departments), no.	312 (5)	452 (10)	469 (10)
Sewershed boundaries, no. (%)‡‡	1,224 (77)	1,233 (82)	1,407 (73)

*IQR, interquartile range; RUCC, Rural-Urban Continuum Code; WWTP, wastewater treatment plant. †Counties were included as long as a wastewater site served some part of the county. ‡By 2023 RUCC (https://www.ers.usda.gov/data-products/rural-urban-continuum-codes). §Some sites are not part of a US Census Region. ¶The US Census Bureau population estimate of 331,449,281 persons (as of April 1, 2020) was used to estimate percent of the US population covered by wastewater sampling sites. That number represents the sum of population served by WWTP sampling locations. Wastewater sewersheds may serve overlapping populations. #Sites may be testing for >1 target; the numbers of sites testing for each target in the table are not mutually exclusive. **Sites may be managed by >1 entity; the numbers of sites managed by each entity in the table are not mutually exclusive. Wastewater data from health departments may reflect testing from public health, commercial, and academic laboratories. ††All samples collected at a site were included in the median of number of samples collected per week regardless of site management. ‡‡Sewershed boundaries are submitted for sites located at a WWTP managed by local or state health departments or the commercial contractor.

The number of sites testing for influenza A virus and RSV increased from 2023 to 2024, whereas total sites across all targets and sites testing for SARS-CoV-2 and MPXV decreased slightly from 2023 to 2024 (Table 1; Figure 1). Influenza A(H5) virus testing in wastewater increased substantially from 2023 to 2024 (25 to 551 sites) as multiple sectors collaborated in a One Health approach to respond to the highly pathogenic avian influenza (HPAI) A(H5N1) outbreak in dairy cattle in 2024 (*9*,*10*).

NWSS also worked with health departments and other partners to increase the coverage of sewershed polygons representing the geographic areas contributing to wastewater sampling sites. In 2024, NWSS received 1,233 sewershed polygons representing 82% of CDC-funded sites. That extensive catalog of geospatial data helps NWSS more accurately define communities served by WS for more effective interpretation of pathogen activity.

## NWSS Sampling and Pathogen Testing

Data for >456,500 samples from 139 laboratories were submitted to NWSS during 2020–2024. Health departments submitted ≈70% of sample data, including testing results generated by state public health, commercial, and academic laboratories (Tables 1, 2). Sample data submitted by the academic partner program peaked at 18% of total samples reported in 2024 and accounted for 12% of samples submitted for the overall analysis period. The median number of samples reported per site per week was 2 (IQR 1–2) across all analysis periods (Table 1).

**Table 2:** Characteristics of samples submitted to the National Wastewater Surveillance System, United States, 2023, 2024, and 2020–2024*

Samples	2023	2024	2020–2024
Total no. samples tested†	155,165	148,023	456,594
Time from sample collection to testing, d, median (IQR)	3 (1–4)	2 (1–4)	2 (1–4)
Time from sample collection to reporting to 1CDP, d, median (IQR)	7 (5–14)	7 (4–10)	8 (5–22)
Time from sample testing to reporting to 1CDP, d, median (IQR)	4 (2–9)	4 (1–7)	5 (2–17)
Sample collection method, no. (%)‡			
Grab	20,170 (13)	20,073 (14)	58,553 (13)
Manual composite	2,382 (2)	926 (<1)	6,106 (1)
Flow-weighted composite	58,552 (38)	57,404 (39)	189,379 (41)
Time-weighted composite	73,031 (47)	68,635 (46)	200,086 (44)
Passive	1,030 (<1)	984 (<1)	2,462 (<1)
Sample type, no. (%)			
Untreated wastewater	116,347 (75)	104,958 (71)	353,935 (78)
Wastewater post–grit removal	30,286 (20)	34,702 (23)	78,122 (17)
Primary effluent	189 (<1)	186 (<1)	465 (<1)
Primary sludge	8,343 (5)	8,177 (6)	24,072 (5)
Sample management, no. (%)§			
Local health department	3,614 (2)	2,745 (2)	10,429 (2)
State health department	102,602 (66)	105,578 (71)	331,293 (72)
Commercial contractor	23,692 (15)	13,413 (9)	58,332 (13)
Academic partner program	25,553 (16)	26,287 (18)	56,993 (12)
PCR type, no. (%)			
qPCR	51,768 (33)	19,656 (13)	145,494 (32)
dPCR	103,114 (66)	127,891 (86)	310,011 (68)
Multiple PCR types used	283 (<1)	476 (<1)	1,089 (<1)
Endogenous wastewater control, no. (%)			
Pepper mild mottle virus	98,542 (64)	96,592 (65)	277,996 (61)
CrAssphage	9,436 (6)	10,049 (7)	30,962 (7)
Other	268 (<1)	201 (<1)	670 (<1)
None	46,919 (30)	41,181 (28)	146,966 (32)
Exogenous wastewater control, no. (%)			
Bovine coronavirus	88,511 (57)	109,731 (74)	270,720 (59)
Bovine respiratory syncytial virus	29,865 (19)	4,418 (3)	58,258 (13)
Other	23,011 (15)	21,821 (15)	82,648 (18)
None	13,778 (9)	12,053 (8)	44,968 (10)
No. samples sequenced for SARS-CoV-2	45,824	40,748	114,394

*1CDP, One CDC Data Platform (https://www.cdc.gov/data-modernization/php/one-cdc-data-platform/index.html); dPCR, digital PCR (including droplet digital PCR); IQR, interquartile range; qPCR, quantitative PCR. †Samples were counted based on unique sample identifications; if a sample was tested for multiple targets, the sample was only counted once. ‡Sample collection methods were determined using the most recent collection-method record associated with each sample. Collection method information may be missing for some samples, and a small number of samples were associated with multiple collection methods. §Sample management counts were not mutually exclusive. Samples could be associated with >1 management category.

Although wastewater sampling and testing methodologies varied across sites and data submitters, >70% of samples were from untreated liquid influent wastewater (Table 2). Liquid influent is easier for most utility operators to sample, whereas other sample types (such as primary sludge) are not available at all treatment plants (*11*,*12*). Of samples collected during 2023 and 2024, >80% were composite samples (e.g., time-weighted, flow-proportional, and manual composite samples). More than two thirds of samples overall were tested using digital or digital droplet PCR, with a notable increase from 2023 (66%) to 2024 (86%). 

Endogenous and exogenous controls are important for validating the accuracy, consistency, and reliability of wastewater testing processes, thereby ensuring high-quality and actionable data (*13*,*14*). In 2023 and 2024, ≈65% of samples tested used pepper mild mottle virus as the endogenous control, whereas no control was used in ≈30% of samples (Table 2). Most samples tested in 2023 and 2024 used bovine coronavirus as the exogenous control organism (57% in 2023 and 74% in 2024) (*14*).

**Figure 3:**
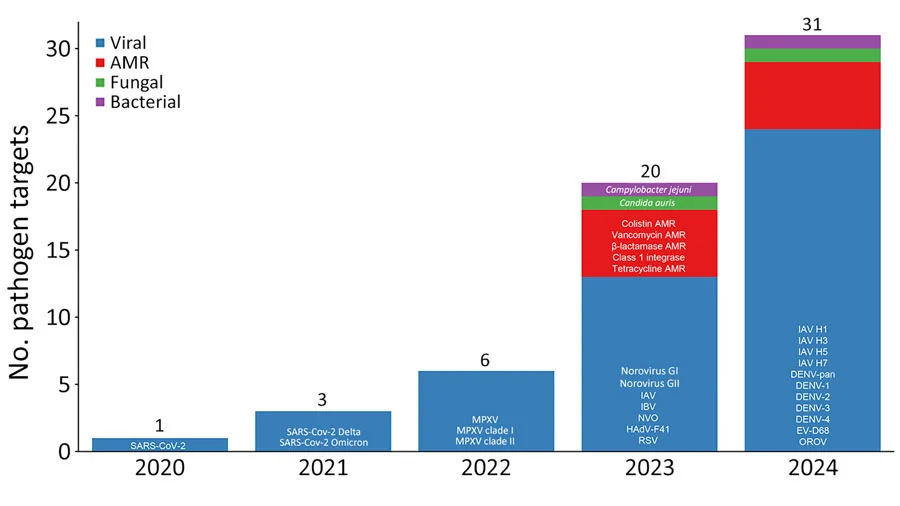
Pathogen targets accepted by the National Wastewater Surveillance System over time, United States, 2020–2024. Stacked bars display pathogen targets for each period, stratified by pathogen target category (viral, fungal, bacterial, and AMR gene targets). Each year’s bar is labeled with the pathogen target(s) added that year. Numbers at tops of bars indicate cumulative number of pathogen targets over time. AMR, antimicrobial resistance; DENV, dengue virus; EV-D68, enterovirus D68; HAdV-F41, human adenovirus type 41 species F; IAV, influenza A virus; IBV, influenza B virus; MPXV, monkeypox virus; NVO, nonvariola orthopoxvirus; OROV, Oropouche virus; pan-DENV, all DENV types; RSV, respiratory syncytial virus.

NWSS accelerated pathogen target ingestion in 2023 and 2024, adding 25 (81%) of 31 total targets to 1CDP and broadening coverage beyond MPXV and SARS-CoV-2 to include bacterial, fungal, other viral, and antimicrobial resistance gene targets (Figure 3). Influenza A virus and RSV were 2 of 14 targets added in 2023, and influenza A(H5) subtype was 1 of 11 targets added in 2024. By expanding its pathogen portfolio, NWSS transformed into a flexible, multipathogen surveillance system supporting both routine and emerging infectious disease surveillance. In addition, the existing 1CDP data infrastructure enabled the rapid integration of new targets through standardized data pipelines and provided a common platform for multipathogen WS used by internal and external partners.

## NWSS Concentration Data Submission, Quality Control, and Timeliness

NWSS collaborates with data submitters to promote timely and accurate data submissions through automation and integrated quality control measures (*15*). Concentration data were ingested into 1CDP through a kill-and-replace method that overwrites the previous upload, enabling easy error correction and backfilling of historical data. Data submitters were encouraged to upload data on a weekly basis (i.e., samples collected during the previous MMWR reporting week) before 11:59 PM on Wednesdays. An automated pipeline in 1CDP flagged data quality issues and reported them via a quality control dashboard available to jurisdictions soon after data submission. NWSS program staff developed additional tools, including submitter-specific reports and pipeline health checks, to triage data quality issues and provide direct technical assistance. We cleaned, parsed, analyzed, and quality-checked wastewater data on Thursdays and made data available for public display and download after successful internal review (*16*–*18*).

Data were submitted to 1CDP via manual upload or automated methods, such as an application programming interface (API) or secure file transfer protocol. All data from academic and national commercial contract partners were submitted via API, whereas most health departments submitted data manually. Automated submissions began with 2 health departments in 2022, expanded to 3 additional health departments in 2023, and added 5 more health departments in 2024, totaling 10 health departments (469 sites) (Table 1).

**Figure 4:**
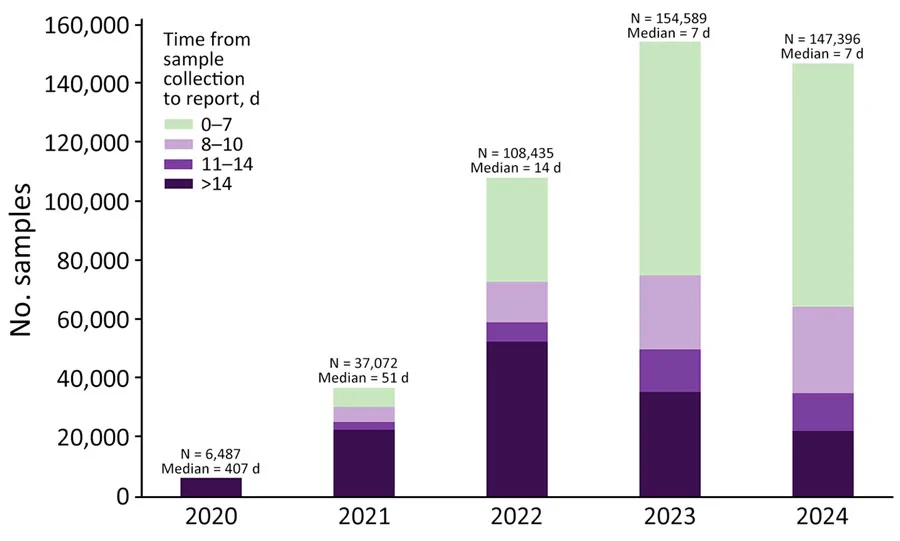
Sample counts and reporting timeliness for the National Wastewater Surveillance System, United States, 2020–2024. Stacked bars display total number of samples submitted during each period stratified by reporting intervals (0–7 days, 8–10 days, 11–14 days, and >14 days from sample collection to reporting). Data include samples tested for SARS-CoV-2, influenza A virus, influenza A(H5) subtype virus, monkeypox virus, and respiratory syncytial virus.

Although the number of samples and pathogen targets submitted to NWSS increased substantially over time, overall reporting times from sample collection to 1CDP data upload improved markedly; the median decreased from 14 days in 2022 to 7 days in 2023 and 2024 (Table 2; Figure 4). Consistent with recommendations from the Association of Public Health Laboratories, samples from the NWSS laboratory network were processed and tested rapidly, resulting in a median time from sample collection to testing of 2–3 days (Table 2) (*13*). The median time from laboratory testing to 1CDP upload was 4 days in 2023 and 2024 (Table 2). Accordingly, in 2023 and 2024, more than half of sample data submitted were reported within 7 days of sample collection, and >75% of sample data were reported within 14 days of collection (Figure 4).

## Wastewater Metric and Data Visualizations Improvements

In 2023, NWSS developed the wastewater viral activity level (WVAL) metric and corresponding categories to standardize and aggregate SARS-CoV-2 data across state, territorial, regional, and national levels and to compare trends over time, replacing the 15-day rolling detection proportion, percentage change, trend, and percentile metrics (*3*,*4*,*19*). For the 2022 MPXV outbreak, we developed a site-level metric to indicate virus detection in samples collected during the previous 4 weeks (*3*,*20*). That metric categorized detections as persistent detection, detection, no recent detection, or no recent data. In 2024, we developed WVALs for influenza A and RSV and created an avian influenza A(H5)–specific metric to classify sites as having a detection, no detection, or no sample tested by reporting week.

WVAL data visualizations on NWSS CDC webpages display historical national, regional, and state-level trends and include national maps showing state- and territory-level activity. Those visualizations enable comparisons of SARS-CoV-2, influenza A, and RSV activity across geographic scales. MPXV and influenza A(H5) visualizations display site-level detection status to support more localized monitoring of potential disease activity. Corresponding data tables are available for all visualizations, and influenza A(H5) includes an additional table displaying detections over a 6-week lookback period to support interpretation of recent detection patterns.

## Wastewater Genomic Sequencing

Wastewater sequencing became a powerful tool for tracking the emergence of genetic SARS-CoV-2 variants over time (*21*). Samples were processed using specialized bioinformatic pipelines that estimate variant proportions in mixed samples, enabling characterization of genetically diverse, mixed populations of multiple co-circulating genomes, rather than determining a single consensus genome, as is common in clinical sequencing analysis (*22*). In 2023 and 2024, approximately half of sites submitted both concentration and sequencing data for SARS-CoV-2 to NWSS, for a total of >86,000 samples (Table 2).

Wastewater whole-genome sequencing at NWSS sites contributed to the early detection and tracking of emerging SARS-CoV-2 variants. NWSS wastewater genomic data demonstrated some of the initial detections of notable lineages, such as initial Omicron variants in 2021, BA.2.86 across multiple US states within weeks of its first identification in August 2023, and JN.1 as it emerged and rapidly attained national predominance in late 2023, paralleling its ascent in clinical genomic surveillance (*23*–*26*). 

## Collaborations and Public Health Capacity Building

The success of NWSS was driven by sustaining collaboration among public and private sector partners and internally with other CDC programs. To strengthen technical capacity across the network, CDC established 4 WS Centers of Excellence (CoEs) in California, Colorado, Houston (Texas), and Wisconsin during 2021–2022 and expanded to 6 CoEs in 2024 with the addition of New York and North Carolina (*27*,*28*). Led by state and local public health departments in partnership with academic institutions and wastewater utilities, the CoEs served as regional hubs for technical assistance, workforce development, applied research, and evaluation to advance WS implementation and innovation. Monthly regional calls organized across the 6 CoE regions provided a forum for jurisdictions to exchange surveillance findings, discuss implementation challenges, share best practices, and enhance regional situational awareness.

CDC incorporated WS into its incident management structure during the 2022 MPXV and 2024 avian influenza A(H5) outbreaks, enabling NWSS data to be integrated with epidemiologic, laboratory, and other surveillance information to inform response activities and public health actions. NWSS worked closely with other CDC programs that provide subject matter expertise for each pathogen target supported by the system, ensuring coordinated interpretation of wastewater signals alongside complementary surveillance data (9,*20*). A prime example of cross-program collaboration was NWSS wastewater data for SARS-CoV-2, influenza A, and RSV being displayed with clinical data on CDC’s public respiratory illness webpages starting in 2024 to provide a more comprehensive understanding of community-level respiratory disease activity (*29*,*30*). Those efforts have strengthened NWSS’s role and utility within CDC’s emergency response and routine surveillance frameworks.

## Discussion

Since its launch in 2020, NWSS has evolved from a rapid response SARS-CoV-2 surveillance pilot into an adaptable, multipathogen surveillance system to support national public health preparedness and response. During 2020–2024, NWSS received wastewater data from >2,300 sites across 53 states and territories, representing 54% of the US population. Although the system achieved broad geographic coverage, site distribution was concentrated in the South and Midwest and included predominantly urban counties. The robust collection of >1,400 sewershed boundary polygons enhanced the geospatial precision of WS catchment areas, enabling improved characterization of communities and community-level indicators that might influence the public health impact of wastewater detections (*31*).

NWSS expanded nationally coordinated surveillance to include influenza A virus, influenza A(H5) virus, RSV, and MPXV and supported surveillance for a total of 31 pathogen targets through 2024, demonstrating the flexibility and scalability of the system to address local and emerging public health priorities. NWSS also strengthened its laboratory and analytic capabilities through increased adoption of digital and digital droplet PCR methods, expansion of wastewater genomic sequencing to support early detection and monitoring of emerging variants, and implementation of pathogen-specific metrics such as the WVAL and associated visualizations. Together, those advancements improved the standardization and interpretation of wastewater data across jurisdictions and over time.

Enhancements to NWSS’s data systems helped improve the timeliness of wastewater concentration data. Greater adoption of automated data submission reduced the time from sample testing to reporting through 1CDP. In addition, code improvements and automated data health checks reduced pipeline run times and improved data quality, further reducing reporting lags. Those improvements have been crucial, given that wastewater signals provide population-level information that can sometimes precede clinical detections and variant identification. That capability supports the utility of NWSS as a public health surveillance system that is independent of health-seeking behavior and testing practices, complements clinical surveillance systems, and can provide early warning of emerging disease activity (*32*,*33*).

The continued growth of NWSS has been supported by sustained collaboration among health departments, wastewater utilities, commercial laboratories, academic institutions, and federal partners. The Wastewater Surveillance CoEs expanded technical capacity, enhanced regional collaboration, and enabled the exchange of best practices across the network. Integration of NWSS into CDC response efforts for mpox and avian influenza A(H5) demonstrated the value of wastewater in monitoring emerging public health threats. NWSS has shown that WS can support coordinated One Health approaches to monitoring zoonotic diseases such as influenza A(H5) while also being sensitive enough to detect emerging infections such as mpox (*9*,*20*,*34*,*35*). 

WS has demonstrated substantial progress as a valuable component of public health infrastructure. Continued development focused on representative coverage, data timeliness, and wastewater sequencing capacities across NWSS will further strengthen its public health utility (*12*,*36*–*39*). Despite NWSS serving ≈178 million persons, coverage gaps persist in some states, rural areas, and unsewered communities. Balancing resource availability with representative coverage and surge capacity needs for emerging threats will remain a critical consideration as jurisdictions continue to optimize WS activities. 

Although reporting times have decreased overall, further reductions could strengthen early responses to emerging infectious disease threats. The overall higher reporting times observed in 2020–2022 were partly the result of newly onboarded partners uploading historical sample results. Timeliness of NWSS data could be improved through faster onboarding of data submitters and earlier submission of data after new assays are validated and implemented. Finally, broader application of wastewater genomic sequencing beyond SARS-CoV-2 to include other emerging pathogens may enhance understanding about community transmission patterns and case-based clinical investigation.

In just over 4 years, NWSS matured into a national surveillance platform that complements traditional case-based and syndromic surveillance and can be leveraged to address national public health threats. NWSS expanded national WS coverage to 5 viral pathogen targets during this period and used its scalable data pipeline architecture to support surveillance of additional viral targets, as well as bacterial, fungal, parasitic, and antimicrobial resistance targets. Strong partnerships with health departments, commercial laboratories, and academic institutions were central to this progress, as were efforts to augment concentration and sequencing data infrastructure to support timely signal interpretation and visualizations for public health decision-making.

Short-term priorities for NWSS include several core system improvements, including optimizing coverage to improve representativeness; increasing standardization of sampling, laboratory, and reporting practices; and scaling up automated data submission to strengthen timeliness and comparability across the network. Future system enhancements will focus on expanding pathogen sequencing capabilities and integrating wastewater data more systematically with clinical and syndromic surveillance data. Sustained funding, workforce capacity building, and continued cross-sector collaboration will be essential to achieving those goals.
